# Influence of Number Location on the SNARC Effect: Evidence From the Processing of Rotated Traditional Chinese Numerical Words

**DOI:** 10.1177/2041669520917169

**Published:** 2020-04-04

**Authors:** Zhiwei Wang, Xiaotong Zhu, Yingjie Jiang

**Affiliations:** School of Psychology, Northeast Normal University, Changchun, China; Xihe Middle School, Chengdu, China; School of Psychology, Northeast Normal University, Changchun, China

**Keywords:** SNARC effect, SRC effect, Simon effect, mental rotation, task

## Abstract

Studies have widely captured the spatial-numerical association of response codes (SNARC) effect in the processing of various types of numbers in which small numbers are responded to faster with the left hand than with the right hand and larger numbers are responded to faster with the right hand than with the left hand. Although a few studies have explored Arabic numbers to further investigate the influence of number location on the SNARC effect, it remains unclear whether the influence of number location on the SNARC effect is moderated by numerical semantic processing difficulty and the task performed. This study explored traditional Chinese numerical words and rotated them to certain angles, which can increase numerical semantic processing difficulty, to further investigate the influence of the stimulus–response compatibility effect and Simon effect on the SNARC effect in a space classification task (Experiment 1), numerical magnitude classification task (Experiment 2), numerical parity classification task (Experiment 3), and color classification task (Experiment 4). The results indicated that (a) the stimulus–response compatibility effect, not the SNARC effect, prevailed in the numerical space classification task; (b) the SNARC effect, not the Simon effect, prevailed in the numerical magnitude and parity classification task; and (c) the Simon effect and the SNARC effect coexisted in the color classification task. These results suggested that the influence of number location on the SNARC effect was moderated by the task performed. Implications for the theory of the SNARC effect and Simon effect are discussed.

Dehaene et al. centrally presented Arabic numbers on a display and asked participants to classify the probe numbers by pressing a specific key according to the numerical magnitude or the numerical parity. The results showed that small numbers were responded to faster with the left key than with the right key, and large numbers were responded to faster with the right key than with the left key, regardless of what classification task was performed. The authors defined this compatibility effect as the spatial-numerical association of response codes (SNARC) effect (Dehaene et al., 1990, 1993). Further studies found that the SNARC effect could be captured not only in the processing of Arabic numbers (Didino et al., 2019; Wang et al., 2018) but also in the processing of other types of symbolic (e.g., French numerical words and Chinese numbers; De Brauwer et al., 2008; Liu et al., 2004) and nonsymbolic numbers (e.g., water quantities, note values, visual illusions, luminance; Fumarola et al., 2014; Kirjakovski & Utsuki, 2012; Prpic et al., 2016, 2018). An increasing number of studies have captured this compatibility effect in the processing of ordinal symbols in which the initial items in ordinal sequences are responded to faster with the left hand than with the right hand and the subsequent items in ordinal sequences are responded to faster with the right hand than with the left hand (Gevers et al., 2003; Previtali et al., 2010; van Dijck & Fias, 2011). Given that a number contains spatial properties and is represented spatially based on its numerical magnitude from left to right (Aiken & Williams, 1973; Galton, 1880; Restle, 1970), Dehaene et al. interpreted this SNARC effect as the result of the mental spatial representation of numbers. Therefore, the SNARC effect is regarded as the gold standard to examine whether a specific symbol is represented spatially in one’s mind, and it has been widely applied in other studies (Shi et al., 2019; Wang et al., 2019; Zhang et al., 2019).

In addition to studies finding that the internal representational space of a specific symbol can influence individual responses, many studies have indicated that the external spatial location of a stimulus deeply influences individual responses. In one study, when participants were asked to classify the external spatial location of specific stimuli by pressing a specific key with the left or right hand, stimuli presented on the left side were responded to faster with the left hand than with the right hand, and stimuli presented on the right side were responded to faster with the right hand than with the left hand. This phenomenon is called the spatial stimulus–response compatibility (SRC) effect (Böffel & Müsseler, 2019; Fitts & Seeger, 1953; Lien & Proctor, 2002; Shi et al., 2020; Yamaguchi et al., 2018). A similar response pattern was found when the task performed was irrelevant to the spatial location of the stimulus and was defined as the Simon effect (Golob & Mock, 2019; Hasbroucq & Guiard, 1991; Medina et al., 2019; Proctor, 2011; Shi et al., 2019; Simon & Small, 1969; Yamaguchi & Proctor, 2019).

Several studies have explored the polarity correspondence principle to interpret the SRC effect, the Simon effect, the SNARC effect, and ordinal position effect (Cho & Proctor, 2003; Cutini et al., 2019; Feldman et al., 2019; Proctor & Cho, 2006; Proctor & Xiong, 2015). According to the polarity correspondence principle, participants can encode a stimulus as a positive polarity or a negative polarity based on the salience of specific attributes (e.g., numerical magnitude, stimulus location). However, they can also encode the response as a positive or a negative polarity. Hence, polarity correspondence leads to the occurrence of these effects in the processing of specific experimental contexts. For example, when participants were asked to perform a numerical magnitude classification task by pressing the left key or right key of the keyboard, they could encode small numbers as a negative polarity and encode large numbers as a positive polarity; meanwhile, they could encode pressing the left key as a negative polarity and pressing the right key as a positive polarity. Hence, the polarity of small numbers was consistent with the polarity of pressing the left key, and the polarity of large numbers was consistent with the polarity of pressing the right key; therefore, the SNARC effect occurred in the processing of numbers (Pinto et al., 2019; Proctor & Cho, 2006; Proctor & Xiong, 2015; Shi et al., 2020).

As the SNARC effect was initially discovered by Dehaene et al. (1993), studies have investigated the mechanism of the SNARC effect from different perspectives (Dodd et al., 2008; Hesse et al., 2016; Shaki et al., 2009; Viarouge et al., 2014; T. Yang et al., 2014). For example, Hesse et al. investigated whether the SNARC effect was moderated by different effectors (fingers, eyes, and arms were used in their study). Their results showed that the SNARC effect could occur in all effectors but varied in strength across the effectors (Hesse et al., 2016). Several studies have also studied the mechanism of the SNARC effect when the spatial location of numbers was induced in experimental contexts (Gevers et al., 2005; Mapelli et al., 2003; Notebaert et al., 2006). For example, Mapelli et al. (2003) randomly presented probe Arabic numbers on the left or right side of the display and asked participants to perform the numerical parity classification task. They indicated that the SNARC effect and the Simon effect could coexist in that experimental context. However, it remains unclear whether the SNARC effect was influenced by the task performed when the spatial location of the numbers was induced. In addition, previous studies on the relation between the SNARC effect and the Simon effect have always explored Arabic numbers as stimuli. The Arabic numbers were very familiar to participants, and thus, Arabic numerals were relatively easy to identify. Several studies on the Simon effect have found that the recognition difficulty of the stimulus has a great influence on the Simon effect (Hasbroucq, 1990; Roswarski & Proctor, 1996; Yamaguchi et al., 2018). For example, one recent study related to the influence of the spatial location of ordinal symbols on the ordinal position effect found that the Simon effect could disappear when participants were asked to perform an ordinal sequence classification task, which increases the difficulty of the cognitive task and hence requires more response time to identify the probe stimulus (Shi et al., 2020). From these studies, we can speculate that if the numerical semantic processing difficulty increases, the influence of the Simon effect on the SNARC effect will change; however, few studies have supported this speculation.

Previous studies have indicated that when participants are asked to identify a rotated stimulus, they rotate the rotated stimulus to the positive position before they identify the rotated stimulus. The greater the rotation angle, the more difficulty participants have in identifying the rotated stimulus (Cooper, 1975; Liesefeld et al., 2015; Shepard & Metzler, 1971; H. Yang & Lupker, 2019). In addition, relevant studies have found that traditional Chinese numerical words are more difficult to recognize than Arabic numbers (Liu et al., 2004). Therefore, in this study, we explored traditional Chinese numerical words as stimuli and rotated them clockwise to certain angles. Then, we randomly presented these words in the left or right space to further investigate the influence of number location on the SNARC effect across different tasks in a context where numerical processing was more difficult than the processing of Arabic numbers.

In Experiment 1, to further investigate how the SRC effect influenced the SNARC effect when the numerical external spatial location is salient, we asked participants to judge whether the probe number was presented on the left or right side (numerical space classification task) by pressing a specific key. In Experiment 2, participants were asked to judge whether the probe number was smaller or larger than five (the numerical magnitude classification task) by pressing the left or right key. In this experiment, we investigated the influence of the Simon effect on the SNARC effect when the numerical magnitude was salient. In Experiment 3, participants were asked to give a key-pressing response for the probe number according to its parity (numerical parity classification task) to investigate whether the Simon effect and the SNARC effect would coexist when the task performed was irrelevant to both the numerical magnitude and the numerical spatial location. In the last experiment, we further asked participants to press a key for the probe numerical words according to color (color classification task). In this experiment, we further investigated whether the difficulty of the task, which was irrelevant to both the numerical magnitude and numerical spatial location, would moderate the influence of the Simon effect on the SNARC effect.

## Experiment 1

In this experiment, to investigate whether the numerical space location could decrease and even impede the SNARC effect, the stimuli were randomly presented on the left or right side of the display, and participants were asked to perform a space classification task.

### Methods

#### Participants

Thirty-six (31 females, *M*_age_ = 18.42 years, standard deviation [*SD*] = 0.996) university students voluntarily participated in the experiment. All participants had normal or corrected-to-normal vision and received a payment of 20 CNY once the experiment was completed. This experiment was approved by the ethics committee of Northeast Normal University. Before all experiments started, we asked participants to sign informed consent documents. The three other experiments were approved, and an informed consent document was signed for each.

#### Stimuli and Apparatus

Eight traditional Chinese numerical words (壹, 贰, 叁, 肆, 陆, 柒, 捌, and 玖, which correspond to 1, 2, 3, 4, 6, 7, 8, and 9) were used as the experimental stimuli. These traditional Chinese numerical words were printed in 72-point Times New Roman font. All stimuli had three rotation angles: 0°, 90°, and 180°. The probe stimuli were randomly presented on the left or right side of a 19″ computer screen (1,024 × 768 resolution and refresh rate 60 Hz). The visual angle of the stimulus between the center of the display and the geometric center of the probe (viewing distance was approximately 50 cm) was 3.38°.

#### Procedure

The experiment was conducted using E-Prime software. The instructions were presented on the display for participants before the experiment started. When the experiment started, a fixation point was first centrally presented on the display and lasted 500 milliseconds. Following fixation, probe stimuli were randomly presented on the left or right side of the display. Once the probe stimuli were presented, participants were asked to judge on which side (left or right) the probe stimulus was presented and give a response by pressing the left key (“F” key) or right key (“J” key) on a QWERTY keyboard as quickly and correctly as possible. Following the participant’s response, a blank screen was presented and lasted 1,500 milliseconds, and then, the next trial started. If the participant did not respond to the probe stimulus within 3 seconds, then the trial was counted as an incorrect response, and the next trial was initiated after a blank screen was displayed. The entire experiment was constructed by two blocks. In one block, the participants were asked to press the left key with their left index finger in response to the left-side stimulus and to press the right key with their right index finger in response to the right-side stimulus. The opposite response pattern was used in the other block. The order of these two blocks was balanced among participants. The entire experiment consisted of 492 trials (480 trials for the formal experiment and 12 practice trials) and lasted approximately 30 minutes.

### Results and Discussion

The reaction times (RTs) were significantly positively related to the error rate across all trials, *r*(36) = .43, *p* < .01, indicating that there was no speed-accuracy trade-off in this experiment. Therefore, the error rate was not subjected to further analyses. The mean RTs (all trials with incorrect responses and trials with RTs beyond three *SD*s were deleted under each treatment, and a total of 2.59% of trials were excluded) were analyzed. A repeated-measures analysis of variance (ANOVA) was conducted with 2 (Response Side: left key vs. right key) × 2 (Numerical Location: left side vs. right side) × 2 (Numerical Magnitude: small numerical words [1, 2, 3, 4] vs. large numerical words [6, 7, 8, 9]) × 3 (Rotation Angle: 0°, 90° vs. 180°) as the within-subject factors.

**Figure 1.**
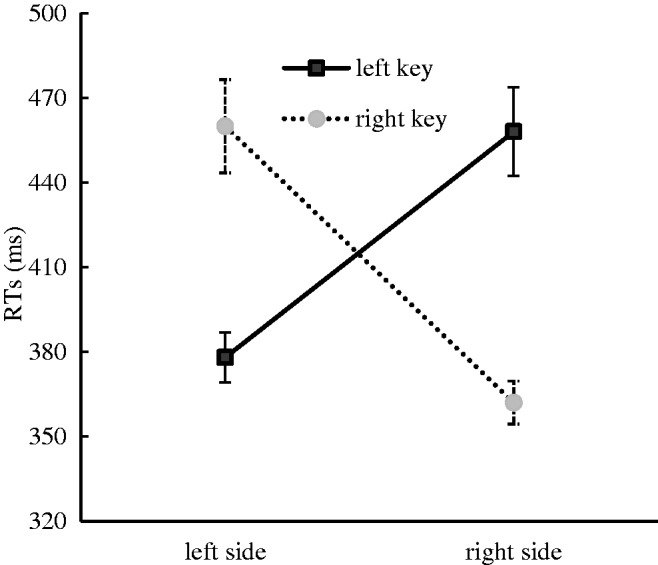
RTs to Stimuli Presented in Different Positions With Different Response Keys for the Space Classification Task in Experiment 1. Error bars represent the standard error. RT = reaction time.

The results indicated that there was a significant main effect for the response side, *F*(1, 35) = 5.39, *p* < .05, η^2^ = .134, and the right key (411 ± 11.78 milliseconds) was responded to faster than the left key (418 ± 11.62 milliseconds). There was also a significant main effect for the numerical location, *F*(1, 35) = 14.52, *p* < .001, η^2^ = .293, in that the numerical words presented on the right side (410 ± 11.20 milliseconds) were responded to faster than the numerical words presented on the left side (419 ± 12.14 milliseconds). This suggested that an advantage effect was captured in the processing of the right spatial stimulus. A significant interaction effect between response side and numerical location was identified, *F*(1, 35) = 75.52, *p* < .001, η^2^ = .683. A further simple effect analysis showed that the numerical words presented on the left side were responded to faster with the left key (378 ± 8.85 milliseconds) than with the right key (460 ± 16.54 milliseconds), *F*(1, 35) = 57.52, *p* < .001, η^2^ = .622, and the numerical words presented on the right side were responded to faster with the right key (362 ± 7.65 milliseconds) than with the left key (458 ± 15.71 milliseconds), *F*(1, 35) = 83.80, *p* < .001, η^2^ = .705. This suggested that the SRC effect occurred in this experiment (Figure 1). No other significant main effects or interaction effects were found in this experiment. In particular, the interaction effect between the response side and the numerical magnitude was not significant, suggesting that the SNARC effect was absent in this experiment.

The space classification task was performed to investigate the influence of the numerical location on the SNARC effect when the numerical location was salient. The results showed that the SRC effect appeared, but the SNARC effect did not. Hence, from these results, we can conclude that stressing the numerical space location can impede the SNARC effect.

## Experiment 2

In this experiment, we aimed to further investigate the influence of the Simon effect on the SNARC effect when the numerical magnitude was salient by asking participants to perform a numerical magnitude classification task.

### Methods

#### Participants

Thirty-six (26 females, *M*_age_ = 18.78 years, *SD* = 1.27) university students voluntarily participated in our experiment. All participants had normal or corrected-to-normal vision and received a payment of 20 CNY once the experiment was completed.

#### Stimuli and Apparatuses

The stimuli and apparatuses used in this experiment were the same as those used in Experiment 1.

#### Procedure

The procedure of Experiment 2 was the same as that used in Experiment 1, with the exception that participants were asked to perform the numerical magnitude classification task in Experiment 2.

### Results and Discussion

There was no significant correlation between RTs and error rate across all trials, *r*(36) = .16, *p* = .35, indicating that there was no speed-accuracy trade-off in this experiment. Therefore, the error rate was not subjected to further analyses. The mean RTs (all trials with incorrect responses and trials with RTs beyond three *SD*s were deleted under each treatment, and a total of 4.52% of trials were excluded) were analyzed. A repeated-measures ANOVA was conducted with 2 (Response Side: left key vs. right key) × 2 (Numerical Location: left side vs. right side) × 2 (Numerical Magnitude: small numerical words [1, 2, 3, 4] vs. large numerical words [6, 7, 8, 9]) × 3 (Rotation Angle: 0°, 90° vs. 180°) as the within-subject factors.

**Figure 2.**
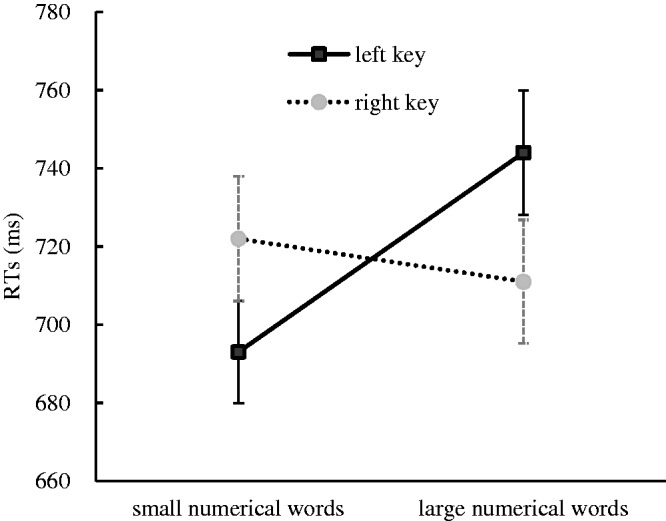
RTs to Small and Large Numerical Words With Different Response Keys for the Numerical Magnitude Classification Task in Experiment 2. Error bars represent the standard error. RT = reaction time.

The results indicated that there was a significant main effect for the numerical magnitude, *F*(1, 35) = 14.45, *p* < .001, η^2^ = .292. Small numerical words (708 ± 13.63 milliseconds) were responded to faster than large numerical words (727 ± 14.82 milliseconds). There was also a significant main effect for the rotation angle, *F*(2, 35) = 61.20, *p* < .001, η^2^ = .636, in that the unrotated numerical words (695 ± 13.21 milliseconds) were responded to faster than the numerical words that were rotated to both 90° (736 ± 14.66 milliseconds) and 180° (721 ± 14.60 milliseconds). This suggested that rotating the numerical words increased the numerical semantic processing difficulty. A significant interaction effect between response side and numerical magnitude was identified, *F*(1, 35) = 10.07, *p* < .01, η^2^ = .223. A further simple effect analysis showed that the small numerical words were responded to faster with the left key (693 ± 13.12 milliseconds) than with the right key (722 ± 15.99 milliseconds), *F*(1, 35) = 7.56, *p* < .01, η^2^ = .178, and the large numerical words were responded to faster with the right key (711 ± 15.81 milliseconds) than with the left key (744 ± 15.90 milliseconds), *F*(1, 35) = 8.80, *p* < .01, η^2^ = .201. This suggested that the SNARC effect occurred in this experiment (Figure 2). A significant interaction effect was also found among the response side, numerical location and numerical magnitude, *F*(1, 35) = 4.80, *p* < .05, η^2^ = .121. This result implied that the interaction between the response side and numerical location, which was viewed as the index of the Simon effect in this experimental context, might be moderated by the numerical magnitude. Therefore, we further analyzed the Simon effect when the presented numbers were smaller than five and when the presented numbers were larger than five. However, the results still did not capture the Simon effect in processing small numerical words, *F*(1, 35) = 0.83, *p* = .369, η^2^ = .023, or large numerical words, *F*(1, 35) = 1.88, *p* = .180, η^2^ = .051. The results further suggested that the Simon effect did not occur in this experiment. No other significant main effects or interaction effects were found in this experiment. In particular, the interaction effect between the response side and the numerical location was not significant, suggesting that the Simon effect was impede in this experiment.

**Figure 3.**
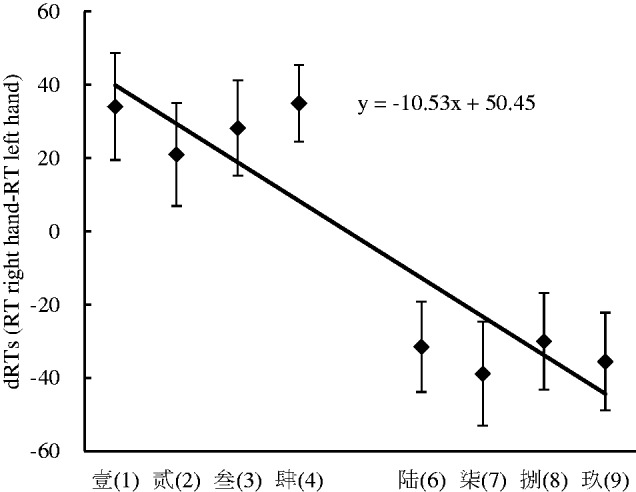
The Linear Regression Plot Between the Number and the dRTs (RT Right Hand Minus RT Left Hand) in Experiment 2. Error bars correspond to the standard error. RT = reaction time.

To further confirm the nature and size of the SNARC effect, regression analysis for dRTs (the RTs of the right hand minus the RTs of the left hand) with numerical words was performed (Fias, 1996; Lorch & Myers, 1990). A linear regression analysis showed that the dRTs decreased by 10.53 milliseconds per numerical word and that the slope coefficient was significantly smaller than zero, *t*(34) = −3.001, *p* < .01, suggesting that the SNARC effect occurred in Experiment 2 (see Figure 3).

The numerical magnitude classification task was performed to investigate the influence of the numerical location on the SNARC effect when the numerical magnitude was salient in Experiment 2. The results showed that the SNARC effect prevailed and the Simon effect did not prevail in this experimental context. It could be concluded from these findings that the activation of the numerical location could not impede the SNARC effect when the numerical magnitude was salient.

## Experiment 3

In this experiment, we aimed to further investigate the influence of the numerical location on the SNARC effect in the numerical parity classification task context in which both the numerical magnitude and the numerical location were irrelevant to the task performed. It should be noted that the numerical words’ semantic information still needed to be deeply processed in this task context.

### Methods

#### Participants

Thirty-six university students voluntarily participated in this experiment. The error rate of one participant was high (21%), and the data from this participant were deleted. The data from the other 35 university students (29 females, *M*_age_ = 20.28 years, *SD* = 2.60) were analyzed. All participants had normal or corrected-to-normal vision and received a payment of 20 CNY once the experiment was completed.

#### Stimuli and Apparatuses

The stimuli and apparatuses used in this experiment were the same as those used in Experiment 1.

#### Procedure

The procedure of Experiment 3 was the same as that used in Experiment 1; however, participants were asked to perform the numerical parity classification task in Experiment 3.

### Results and Discussion

There was no significant correlation between RTs and error rate across all trials, *r*(35) = −.07, *p* = .678, indicating that there was no speed-accuracy trade-off in this experiment. Therefore, the error rate was not subjected to further analyses. The mean RTs (all trials with incorrect responses and trials with RTs beyond three *SD*s were deleted under each treatment, and a total of 6.10% of trials were excluded) were analyzed. A repeated-measures ANOVA was conducted with 2 (Response Side: left key vs. right key) × 2 (Numerical Location: left side vs. right side) × 2 (Numerical Magnitude: small numerical words [1, 2, 3, 4] vs. large numerical words [6, 7, 8, 9]) × 3 (Rotation Angle: 0°, 90° vs. 180°) as the within-subject factors.

**Figure 4.**
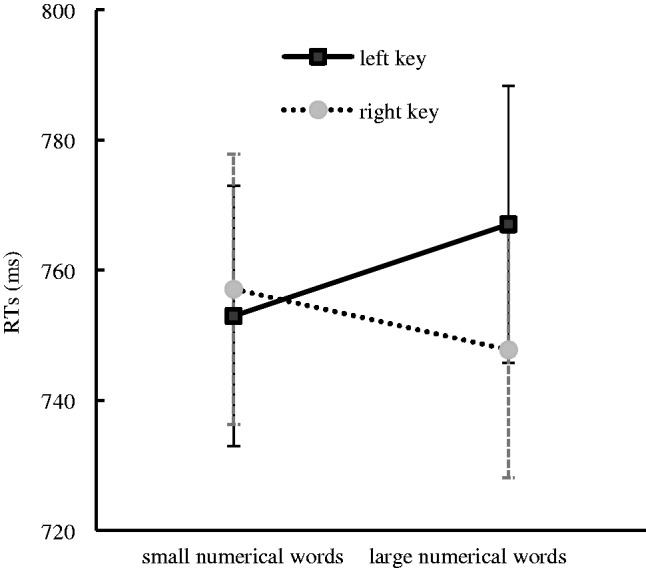
RTs to Small and Large Numerical Words With Different Response Keys for the Numerical Parity Classification Task in Experiment 3. Error bars represent the standard error. RT = reaction time.

The results indicated that there was a significant main effect for the rotation angle, *F*(2, 34) = 36.72, *p* < .001, η^2^ = .519, in that the unrotated numerical words (731 ± 18.67 milliseconds) were responded to faster than the numerical words that were rotated to both 90° (766 ± 20.16 milliseconds) and 180° (772 ± 21.32 milliseconds). This suggested that rotating the numerical words increased the recognition difficulty. A significant interaction effect between response side and numerical magnitude was identified, *F*(1, 34) = 5.35, *p* < .05, η^2^ = .136. A further simple effect analysis showed that there was no significant difference in RTs in the processing of the small numerical words between the left key (753 ± 20.002 milliseconds) and the right key (757 ± 20.777 milliseconds), *F*(1, 34) = 0.596, *p* = .446, η^2^ = .017, and the large numerical words were responded to faster with the right key (748 ± 19.69 milliseconds) than with the left key (767 ± 21.31 milliseconds), *F*(1, 34) = 5.09, *p* < .05, η^2^ = .13. This suggested that the SNARC effect occurred in this experiment (Figure 4). No other significant main effects or interaction effects were found in this experiment. In particular, the interaction effect between the response side and the numerical location was not significant, suggesting that the Simon effect was not impede in this experiment.

**Figure 5.**
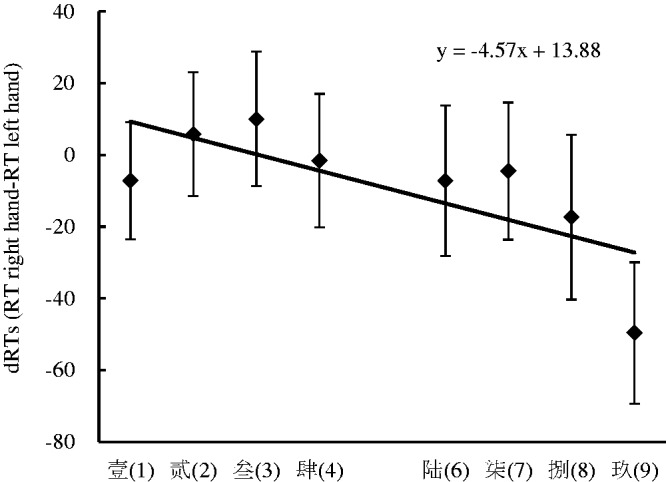
The Linear Regression Plot Between the Number and the dRTs (RT Right Hand Minus RT Left Hand) in Experiment 3. Error bars correspond to the standard error. RT = reaction time.

To further confirm the nature and size of the SNARC effect, the same linear regression analysis as that used in Experiment 2 was performed. The results showed that the dRTs decreased by 4.57 milliseconds per numerical word and that the slope coefficient was significantly smaller than zero, *t*(35) = −2.14, *p* < .05, suggesting that the SNARC effect occurred in Experiment 3 (see Figure 5).

The numerical parity classification task was performed to investigate the influence of the numerical location on the SNARC effect when the numerical parity was salient in Experiment 3. The results showed that the SNARC effect prevailed and the Simon effect was absent in this experimental context. It could be concluded from these findings that the activation of numerical location did not impede the SNARC effect even when the numerical parity was salient.

## Experiment 4

In this experiment, we aimed to further investigate whether the Simon effect and the SNARC effect would coexist in the color classification task context in which both the numerical magnitudes and the numerical location were irrelevant to the task performed. In addition, the numerical words’ semantic information was not needed for deep processing.

### Methods

#### Participants

Thirty-six university students (28 females, *M*_age_ = 18.19 years, *SD* = 0.58) voluntarily participated in this experiment. All participants had normal or corrected-to-normal vision and received a payment of 20 CNY once the experiment was completed.

#### Stimuli and Apparatuses

The stimuli and apparatuses used in this experiment were the same as those used in Experiment 1, with the exception that all of the traditional Chinese numerical words were colored green and black.

#### Procedure

The procedure of Experiment 4 was the same as that used in Experiment 1; however, participants were asked to perform the color classification task in Experiment 4.

### Results and Discussion

There was no significant correlation between RTs and error rate across all trials, *r*(36) = .19, *p* = .265, indicating that there was no speed-accuracy trade-off in this experiment. Therefore, the error rate was not subjected to further analyses. The mean RTs (all trials with incorrect responses and trials with RTs beyond three *SD*s were deleted under each treatment, and a total of 3.38% of trials were excluded) were analyzed. A repeated-measures ANOVA was conducted with 2 (Response Side: left key vs. right key) × 2 (Numerical Location: left side vs. right side) × 2 (Numerical Magnitude: small numerical words [1, 2, 3, 4] vs. large numerical words [6, 7, 8, 9]) × 3 (Rotation Angle: 0°, 90° vs. 180°) as the within-subject factors.

**Figure 6.**
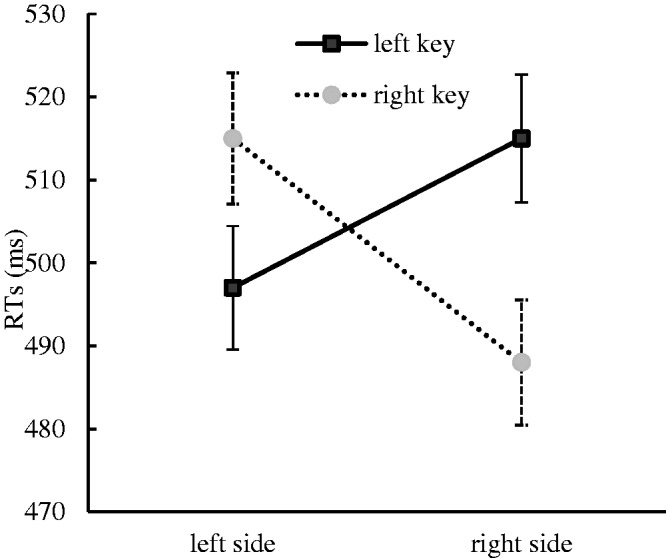
RTs to Stimuli Presented on Different Positions With Different Response Keys for the Color Classification Task in Experiment 4. Error bars represent the standard error. RT = reaction time.

**Figure 7.**
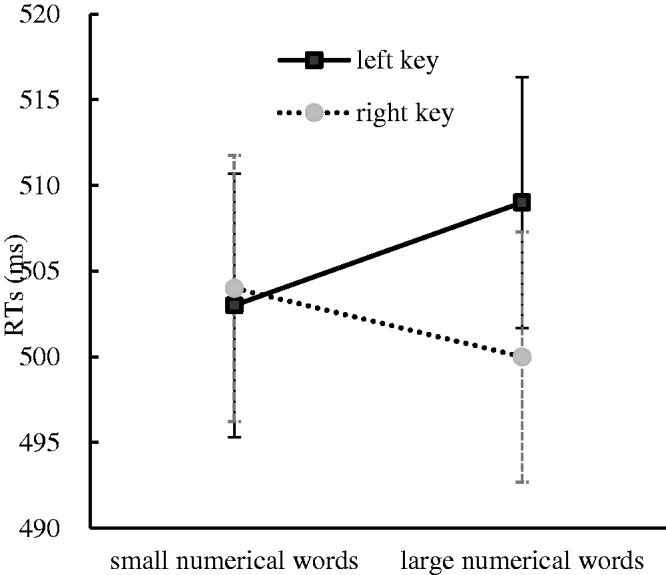
RTs to Small and Large Numerical Words With Different Response Keys for the Color Classification Task in Experiment 4. Error bars represent the standard error. RT = reaction time.

The results indicated that the interaction effect between response side and numerical location was significant, *F*(1, 35) = 49.17, *p* < .001, η^2^ = .584. A further simple effect analysis showed that the numerical words presented on the left side were responded to faster with the left key (497 ± 7.45 milliseconds) than with the right key (515 ± 7.89 milliseconds), *F*(1, 35) = 17.04, *p* < .001, η^2^ = .327, and the numerical words presented on the right side were responded to faster with the right key (488 ± 7.57 milliseconds) than with the left key (515 ± 7.73 milliseconds), *F*(1, 35) = 22.17, *p* < .001, η^2^ = .388. This suggested that the Simon effect occurred in this experiment (Figure 6). A significant interaction effect between response side and numerical magnitude was also identified, *F*(1, 35) = 6.41, *p* < .05, η^2^ = .155. A further simple effect analysis showed that there was no significant difference in RTs in the processing of the small numerical words between the left key (503 ± 7.69 milliseconds) and the right key (504 ± 7.77 milliseconds), *F*(1, 35) = 0.008, *p* = .929, η^2^ = .000, and the large numerical words were responded to faster with the right key (500 ± 7.30 milliseconds) than with the left key (509 ± 7.31 milliseconds), *F*(1, 35) = 4.89, *p* < .05, η^2^ = .123. This suggested that the SNARC effect occurred in this experiment (Figure 7). No other significant main effects or interaction effects were found in this experiment.

**Figure 8.**
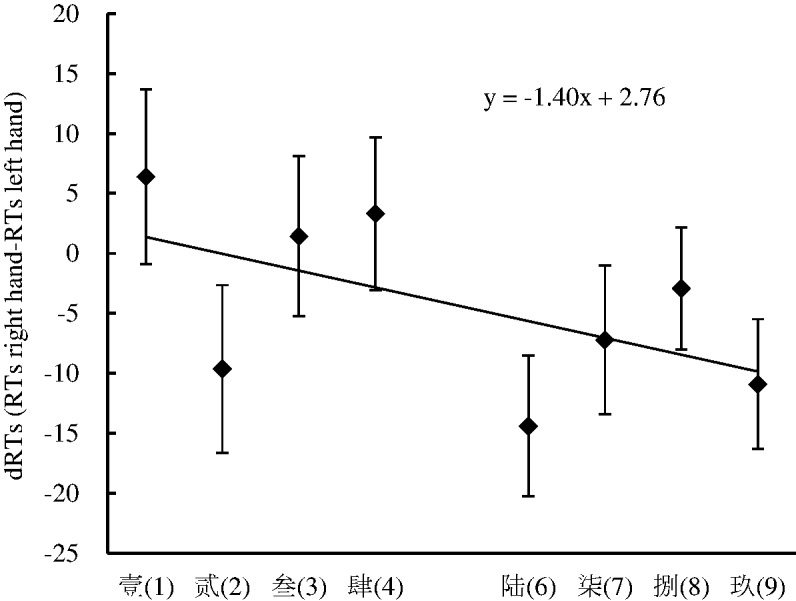
The Linear Regression Plot Between the Number and the dRTs (RT Right Hand Minus RT Left Hand) in Experiment 4. Error bars correspond to the standard error. RT = reaction time.

To further confirm the nature and size of the SNARC effect, the same linear regression analysis as that used in Experiment 2 was performed. The results showed that the dRTs decreased by 1.40 milliseconds per numerical word and that the slope coefficient was significantly smaller than zero, *t*(35) = −1.40, *p* < .05, suggesting that the SNARC effect occurred in Experiment 3 (see Figure 8).

The numerical word color classification task was performed to investigate the influence of the numerical location on the SNARC effect when the numerical color was salient in Experiment 4. The results showed that the SNARC effect and the Simon effect coexisted in this experimental context.

## Discussion

Although previous studies have explored Arabic numbers to investigate the influence of numerical location on the SNARC effect, it remains unclear how numerical semantic processing difficulty and cognitive tasks moderate the influence of numerical location on the SNARC effect. Therefore, this study manipulated the numerical semantic processing difficulty by exploring traditional Chinese numerical words and rotating them to certain angles as stimulus numbers to further investigate the influence mechanism of the numerical location on the SNARC effect across different classification task contexts.

In the first experiment, the participants were asked to perform a spatial location classification task when the rotated traditional Chinese numerical words were presented on the left or right side. The results indicated that the SRC effect occurred, but the SNARC effect was not captured in this experimental context. Previous studies have consistently found that the SNARC effect can be captured in the processing of symbolic and nonsymbolic numbers, including traditional Chinese numerical words (De Brauwer et al., 2008; Didino et al., 2019; Fumarola et al., 2014; Kirjakovski & Utsuki, 2012; Liu et al., 2004; Prpic et al., 2016, 2018). In Experiment 1, when the rotated traditional Chinese numerical words were presented on the left or right side and the spatial location classification task was performed, the SNARC effect did not occur. These results suggest that the salience of the spatial location of traditional Chinese numerical words could impede the SNARC effect. The reason may be that when the spatial location classification task was performed, the numerical semantic information could not be activated; therefore, the SNARC effect was absent. This interpretation was also supported by the absence of a main effect of the rotation angles in Experiment 1.

In the second experiment, the probe stimulus was presented in the same way as in Experiment 1, but the numerical magnitude classification task was performed in Experiment 2. The result showed that the SNARC effect prevailed and the Simon effect did not occur in this experimental context. The SNARC effect stably and universally occurred in the processing of all types of numbers, so when the numerical magnitude classification task was performed, the SNARC effect prevailed. However, although most studies have indicated that the Simon effect occurs when the experimental task is irrelevant to the location of the spatial stimulus, several studies have found that the magnitude of the Simon effect decreases with increasing response times (Hasbroucq & Guiard, 1991; Proctor et al., 2009; Shi et al., 2020). In Experiment 2, participants performed the numerical magnitude classification task, which was irrelevant to the spatial location of the traditional Chinese numerical words, but the Simon effect did not occur. The reason might be that the semantic information of the traditional Chinese numerical words was more difficult to process than that of the Arabic numbers, and rotating traditional Chinese numerical words further increased the numerical semantic processing difficulty. The increased numerical processing difficulty further increased the response times to the probe stimulus and thus impede the Simon effect.

In Experiment 3, we further asked participants to complete a numerical parity classification task, which was irrelevant to both the numerical spatial location and numerical magnitude and needed to be directly processed for the numerical words’ semantic information. The results of Experiment 3 showed that the SNARC effect prevailed and the Simon effect disappeared. Previous studies explored Arabic numbers to investigate the influence of the Simon effect on the SNARC effect, and the results showed that the Simon effect and the SNARC effect could coexist when the numerical parity classification task was performed (Gevers et al., 2005; Mapelli et al., 2003; Notebaert et al., 2006). In Experiment 3, the numerical parity classification task was performed, but the Arabic numbers were replaced by traditional Chinese numerical words. The results showed that the SNARC effect prevailed and that the Simon effect did not. The reason may be that numerical semantic processing is more difficult for traditional Chinese numerical words than for Arabic numbers. These results suggested that whether the SNARC effect and the Simon effect could coexist in the numerical parity classification task was moderated by the numerical attributes. In particular, when the semantic processing of the probe numbers was relatively easy, the SNARC effect and Simon effect could coexist in the numerical parity classification task. However, when the semantic processing of the probe numbers was relatively difficult, the SNARC effect prevailed and the Simon effect could not occur in the numerical parity classification task.

If the Simon effect of Experiment 2 and Experiment 3 disappeared because of the semantic processing difficulty of the traditional Chinese numerical words, the Simon effect would occur in the traditional Chinese numerical words’ semantic information irrelevant task condition because in this task condition, the traditional Chinese numerical words’ semantic information did not need to be processed directly. Therefore, we used the color classification task to further investigate the influence of the Simon effect on the SNARC effect in Experiment 4. The results showed that the SNARC effect and the Simon effect coexisted when the color classification task was performed. Previous studies have found that the semantic information of a stimulus can be automatically processed when participants identify the stimulus’ colors (Cohen et al., 1990; Grégoire et al., 2019). When the color classification task was performed in Experiment 4, the Chinese numerical words’ semantic information could be more or less processed; therefore, the SNARC effect was nonsignificant. It should be noted that when the color classification task was performed, the task was completed even when the numerical semantic information was not processed; however, the numerical semantic information had to be clearly processed when the numerical parity classification task was performed, although both the numerical color classification and the numerical parity classification task were irrelevant to the numerical magnitude and the numerical spatial location. Therefore, the processing response times were longer in the numerical parity classification task than in the color classification task. Hence, the Simon effect occurred in the numerical color classification in Experiment 4, but it did not occur in the numerical parity classification task in Experiment 3. Previous studies explored Arabic numbers to investigate the influence of the Simon effect on the SNARC effect, and the results showed that the Simon effect and the SNARC effect could coexist when the numerical parity classification task, which was irrelevant to both the numerical magnitude and the numerical spatial location, was performed (Gevers et al., 2005; Mapelli et al., 2003; Notebaert et al., 2006). These results further suggested that whether the SNARC effect and the Simon effect could coexist in numerical magnitude and numerical spatial location-irrelevant tasks depended on the attributes of the task performed. In particular, when the numerical magnitude and numerical spatial location-irrelevant tasks performed were simple and did not require longer response times, the Simon effect and the SNARC effect could coexist. However, when the numerical magnitude and numerical spatial location-irrelevant tasks performed were relatively difficult and required longer response times, the SNARC effect prevailed and the Simon effect disappeared.

Several studies on the SNARC effect found that the size of the SNARC effect was moderated by semantic processing difficulty, and the higher the response time in numerical processing, the larger the size of the SNARC effect (Gevers et al., 2006; Wood et al., 2008). Moreover, several studies found that the size of the Simon effect decreased with increasing response times (Hasbroucq & Guiard, 1991; Proctor et al., 2009; Shi et al., 2020). In Experiments 2 and 3 of this study, the results showed that the response times to the rotated Chinese numerical words were significantly longer than those to the unrotated Chinese numerical words; however, both the SNARC effect and the Simon effect were not influenced by the rotation angles of the Chinese numerical words. The reason might be that the semantic processing difficulty of the traditional Chinese number words was so great that the ceiling effect occurred in these experiments; therefore, the influence of response times on the SNARC effect and on the Simon effect was impede.

In addition, several researchers have indicated that the polarity correspondence between stimulus encoding and response encoding leads to the occurrence of the SNARC effect and the Simon effect in the processing of specific experimental contexts (Cho & Proctor, 2003; Proctor & Cho, 2006; Proctor & Xiong, 2015). In addition, whether participants encode a stimulus as a positive polarity or a negative polarity is based on the salience of specific attributes (e.g., numerical magnitude, stimulus location). In Experiment 1, the spatial location task was performed; therefore, the spatial location was salient, and the results captured the Simon effect. In Experiments 2 and 3, numerical semantic information was processed, and therefore, semantic information was salient. The results showed that the SNARC effect prevailed, and the Simon effect was absent. These results support the prediction of polarity correspondence theory. Furthermore, both the SNARC effect and the Simon effect were present in the color classification task of the traditional Chinese number words, implying that participants could encode the polarity of the stimulus based on the two different dimensions of the stimulus simultaneously.

## Conclusion

From this study, we can conclude that whether the SNARC effect can coexist with the SRC effect or the Simon effect is moderated by the task performed.
